# Metformin add-on continuous subcutaneous insulin infusion on precise insulin doses in patients with type 2 diabetes

**DOI:** 10.1038/s41598-018-27950-9

**Published:** 2018-06-26

**Authors:** Feng-fei Li, Bing-li Liu, Guo-ping Yin, Reng-na Yan, Dan-feng Zhang, Jin-dan Wu, Lei Ye, Xiao-fei Su, Jian-hua Ma

**Affiliations:** 1 Department of Endocrinology, Nanjing First Hospital, Nanjing Medical University, Nanjing, China; 2 National Heart Research Institute Singapore, National Heart Centre, Singapore, Singapore

## Abstract

To investigate whether metformin add-on to the continuous subcutaneous insulin infusion (Met + CSII) therapy leads to a significant reduction in insulin doses required by type 2 diabetes (T2D) patients to maintain glycemic control, and an improvement in glycemic variation (GV) compared to CSII only therapy. We analyzed data from our two randomized, controlled open-label trials. Newly diagnoses T2D patients were randomized assigned to receive either CSII therapy or Met + CSII therapy for 4 weeks. Subjects were subjected to a 4-day continuous glucose monitoring (CGM) at the endpoint. Insulin doses and GV profiles were analyzed. The primary endpoint was differences in insulin doses and GV between the two groups. A total of 188 subjects were admitted as inpatients. Subjects in metformin add-on therapy required significantly lower total, basal and bolus insulin doses than those of control group. CGM data showed that patients in Met + CSII group exhibited significant reduction in the 24-hr mean amplitude of glycemic excursions (MAGE), the standard deviation, and the coefficient of variation compared to those of control group. Our data suggest that metformin add-on to CSII therapy leads to a significant reduction in insulin doses required by T2D patients to control glycemic variations.

## Introduction

Since the emergence of continuous subcutaneous insulin infusion (CSII) therapy in 1997^1^, CSII has become the potential treatment for type 2 diabetes (T2D). Early implementation of a 2 to 3-week course of intensive insulin therapy leads to a prolonged glycemic control in newly diagnosed T2D^2^. The mechanisms by which CSII achieves protracted glycemic remission in T2D may be the improvement in beta-cell function and insulin resistance^1–4^, and, may partially, because the positive attitudes towards diabetes by CSII therapy^5^. In addition, despite the fact that CSII lowers blood glucose concentrations, the incidence of severe hypoglycemia is rare^6,7^. Patients with longstanding T2D, even previous use of intensive insulin therapy, still have favorable outcomes on the mean glycated hemoglobin (HbA_1c_) and glycemic variations^8^. Using continuous glucose monitoring (CGM), we also demonstrated that treatment with CSII provided a greater improvement in blood glucose fluctuations in newly diagnosed or longstanding T2D patients^9^.

Patients with high blood glucose fluctuations may have some risks for long-term diabetic complications^10,11^. To decrease the clinical implications, patients with T2D should be treated in a manner aimed at reducing glycemic variations, especially acute glucose swings, and HbA1c values^12^. CGM provides the potential to mitigate glycemic variations (GV) in subjects with diabetes^13–16^. Higher GV, namely 24-hr standard deviation of the MG (SD) and the 24-hr mean amplitude of glycemic excursions (MAGE), was verified even in subjects with prediabetes^17^. Studies have also indicated that SD and MAGE have strong correlations^13,18^. The percent coefficient of variation (%CV) may be a good indicator of GV^13,18,19^. Using CGM data, physicians can efficiently address the larger blood glycemic variations, which might be important for decision-making^20^.

Metformin is a safe insulin-sparing diabetes agent^21^, and was established as the first-line therapy for T2D. By enhancing insulin sensitivity in the liver and muscle, metformin exerts a glucose-lowering efficacy^22–25^. A trial conducted in subjects with type 1 diabetes indicated that a metformin add-on CSII therapy resulted in an improvement in blood glucose control and insulin requirements^21^. In patients with T2D, the metformin combination with CSII significantly decreased the time required to achieve euglycemic control, the daily insulin dose, the insulin resistance, and increased β cell function^26^. The study also demonstrated that a NPH plus metformin treatment can improve mean blood glucose (MBG), MAGE, and SD^27^.

It is unknown whether a metformin add-on to the CSII therapy leads to a significant reduction in insulin doses required by T2D patients to maintain glycemic control, and an improvement in glycemic variation in the hospital setting compared to CSII only therapy. We previously performed two clinical trials [ClinicalTrials.gov, CliCTR-TRC-10001218 Registration date 13^th^ Feb. 2011, and ClinicalTrials.gov, number NCT03226210 Registration date: 19^th^ Jul. 2017] on newly diagnosed T2D patients treated with CSII with or without metformin add-on therapy to assess GV monitored by CGM^28–30^. In the present paper, we have analyzed the results according to insulin doses and GV, and we report that newly diagnosed T2D patients with metformin add-on to CSII therapy required significantly lower insulin doses to maintain euglycemic control compared to those with CSII therapy only.

## Results

### Baseline characteristics in patients between the two groups

A total of 188 patients were recruited for the study. At the conclusion of the study, patients in the metformin add-on CSII therapy and CSII only therapy group numbered 95 and 93, respectively. There were no significant demographic differences in terms of mean values age, body-mass index, fasting plasma glucose concentrations, fasting plasma insulin concentrations, HOMA-IR, HOMA-B, Insulinogenic index, and Matsuda index between groups at baseline (Table 1).

**Table 1:** Characteristics in patients at baseline between the two groups.

Items	Metformin + CSII	CSII	P
N	95 (58 Male)	93 (50 Male)	/
Age (years)	52.32 ± 8.24	50.17 ± 11.57	0.20
BMI (Kg/m^2^)	24.42 ± 3.27	25.15 ± 3.16	0.18
FPG (mmol/L)	11.70 ± 2.72	11.57 ± 2.76	0.77
FPI (mU/L)	5.74 ± 5.10	6.71 ± 3.27	0.19
FC-P (ng/mL)	1.98 ± 0.73	2.19 ± 0.78	0.12
HOMA-IR	3.25 ± 2.09	3.54 ± 1.87	0.43
HOMA-B	12.92 ± 7.86	11.05 ± 10.43	0.24
Insulinogenic Index	8.28 ± 5.47	10.14 ± 10.28	0.29
Matsuda Index	5.44 ± 2.67	4.63 ± 2.42	0.08
HbA_1c_ (%)	9.67 ± 1.34	8.84 ± 1.38	0.13

BMI: Body mass index, FPG: Fasting plasma glucose concentrations, FPI: Fasting plasma insulin concentrations, FC-P: Fasting C-peptide concentrations.

### Glycemic control and insulin profiles between groups

Patients in metformin add-on group reached their glycemic goals quicker than the control group did (3.97 ± 1.81 vs. 4.84 ± 2.63 days, P < 0.05). The daily total insulin dose required by subjects to maintain euglycemic control in the metformin add-on CSII therapy group was significantly lower than that required by the control group at the conclusion of the study (0.58 ± 0.28 vs. 0.28 ± 0.13 U/Kg, P < 0.01). We analyzed the bolus insulin doses and the basal insulin doses used by patients between groups. Our data showed that patients in metformin add-on CSII group needed significantly lower basal insulin doses (0.31 ± 0.16 vs. 0.17 ± 0.08 U/Kg, P < 0.01) and bolus insulin doses (0.26 ± 0.18 vs. 0.11 ± 0.08 U/Kg, P < 0.01) than those in the control group to maintain glycemic control.

### Glycemic profiles

**Figure 1:**
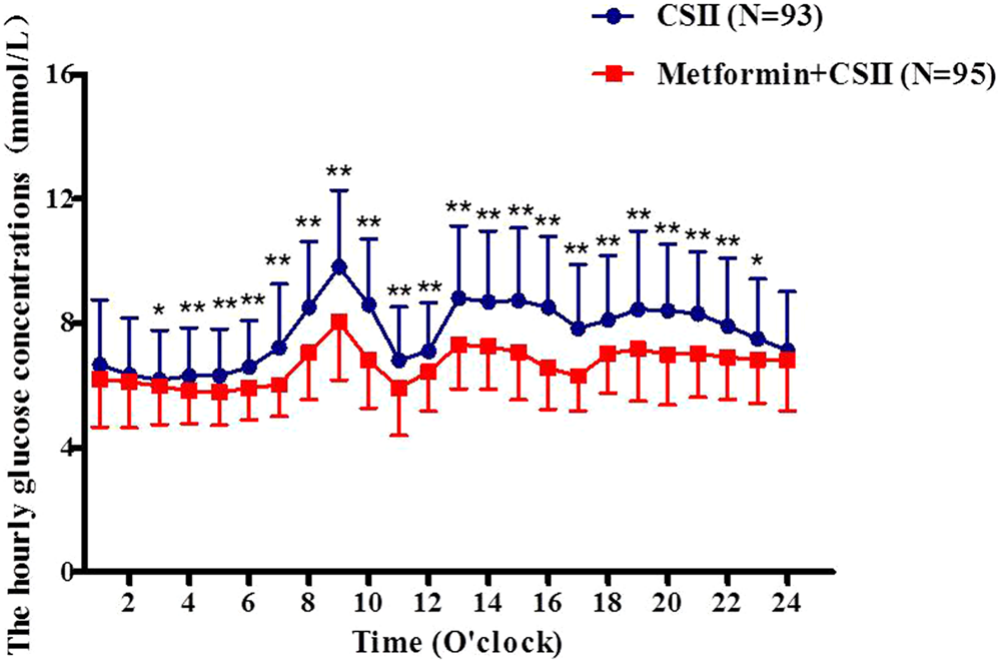
The hourly glucose concentrations in patients between the two groups,*compared with control Group (P < 0.05).

Our CGM data showed that the hourly glucose concentrations in patients treated with metformin combined with CSII therapy were significantly lower than those of control group, especially from 0400 to 2300 o’clock (P < 0.05, respectively) (Fig. 1). Furthermore, we also analyzed the 0000–0600, 0000–0300 and 0300–0600 hours glucose concentrations between the two groups at the conclusion of the study. Our data showed that patients treated with the metformin add-on CSII therapy had significantly lower glucose concentrations during 0300–0600 hours (5.84 ± 0.96 vs. 6.40 ± 1.45 mmol/L, P < 0.05), although patients had similar glucose concentrations during 0000–0600 and 0000–0300 hours (5.97 ± 1.07 vs. 6.40 ± 1.54 mmol/L and 6.10 ± 1.37 vs. 6.40 ± 1.78 mmol/L, P > 0.05, respectively) between the two groups.

Our data also showed that patients in the metformin add-on therapy group had significant reduction in 24-hr MG (6.62 ± 0.78 vs. 7.89 ± 1.62 mmol/L, P < 0.01), the MAGE (3.35 ± 1.71 vs. 4.62 ± 1.80 mmol/L, P < 0.01), the SD (1.33 ± 0.51 vs. 1.92 ± 0.71 mmol/L, P < 0.01), the CV (18.6 ± 6.6 vs. 22.8 ± 6.2%, P < 0.01), the incremental of AUC of glucose >10.0 mmol/L (0.04 ± 0.09 vs. 0.16 ± 0.16 mmol/L*Day, P < 0.01), the 2-hr postprandial glucose levels after three meals (after breakfast: 7.55 ± 1.62 vs. 9.16 ± 2.08 mmol/L, after lunch: 6.88 ± 1.26 vs. 7.96 ± 1.71 mmol/L, after dinner: 7.10 ± 1.33 vs. 8.28 ± 2.10 mmol/L, P < 0.01, respectively) than those of control group. We did not observe any difference in the incremental AUC of glucose less than 3.9 mmol/L between the two groups (0.01 ± 0.03 vs. 0.01 ± 0.02 mmol/L*Day, P > 0.05) (Table 2).

**Table 2:** Changes in glycemic control in patients between the two groups after therapy.

Items	CSII	Metformin + CSII	P
24-hr MG	7.89 ± 1.62	6.62 ± 0.78	0.00
SD	1.92 ± 0.71	1.33 ± 0.51	0.00
CV	22.8 ± 6.2	18.6 ± 6.6	0.00
MAGE	4.62 ± 1.80	3.35 ± 1.71	0.00
AUC > 10	0.16 ± 0.16	0.04 ± 0.09	0.00
AUC < 3.9	0.01 ± 0.02	0.01 ± 0.03	0.71
0–6-hr MG	6.40 ± 1.54	5.97 ± 1.07	0.06
0–3-hr MG	6.40 ± 1.78	6.10 ± 1.37	0.26
3–6-hr MG	6.40 ± 1.45	5.84 ± 0.96	0.01
2-hr PBMG	9.16 ± 2.08	7.55 ± 1.62	0.00
2-hr PLMG	7.96 ± 1.71	6.88 ± 1.26	0.00
2-hr PDMG	8.28 ± 2.10	7.10 ± 1.33	0.00

24-hr MG: 24-hr mean glucose (mmol/L), SD: standard deviation (mmol/L), CV: coefficient of variation (%), MAGE: mean amplitude of glycemic excursions (mmol/L), AUC >10: the incremental area under curve of glucose >10.0 mmol/L (mmol/L*Day), AUC < 3.9: the incremental area under curve of plasma glucose < 3.9 mmol/L (mmol/L*Day), 0–6-hr MG: 0000–0600 o’clock mean glucose (mmol/L), 0–3-hr MG: 0000–0600 o’clock mean glucose (mmol/L), 3–6-hr MG: 0300–0600 o’clock mean glucose (mmol/L), 2-hr PBMG: 2-hr post breakfast mean glucose (mmol/L), 2-hr PLMG: 2-hr post lunch mean glucose (mmol/L), 2-hr PDMG: 2-hr post dinner mean glucose (mmol/L).

We analyzed dawn phenomenon in this study, the incidence of dawn phenomenon was 3.1% (6 out of 195 subjects). The average magnitude of the dawn phenomenon was 2.60 ± 2.20 mmol/L, and the percentage of their nights do people display this phenomenon is 21.1%.

### Safety and tolerance

A total of 16 patients in metformin add-on CSII therapy group had mild digestive disorders. We also compared the risk of severe hypoglycemia (glucose level <3.9 mmol/L) between the two groups. Although Table 2 showed the incremental of area under the curve of glucose concentration less than 3.9 mmol/L was similar between the two groups. The hypoglycemic events in CSII group were statistically significant higher compared to those in Metformin add-on CSII group (17 vs. 7 patients, *P* = 0.03).

## Discussion

We analyzed data from our two randomized, controlled open-label trials on newly diagnoses T2D patients randomized assigned to receive either CSII therapy or metformin add-on to CSII therapy. Our data demonstrated that a metformin add-on to the CSII therapy can significantly reduce the total daily insulin doses to maintain euglycemic control and facilitate further improvements in glycemic variations. We also found that patients treated with the combined metformin and CSII therapy needed a shorter treatment period to achieve euglycemic control than those in the control (CSII) group at the end of the study, and that combination therapy resulted in no increase of hypoglycemia.

Metformin, a family number of biguanide, has become established as the first-line treatment in management of T2D. In combination with insulin in treatment of T2D, the use of metformin leads to the increase in insulin sensitivity and the reduction of daily insulin doses^22–24^. In this study, we observed that patients in the metformin add-on CSII therapy achieved euglycemic control in significantly shorter times, and with lower daily insulin doses than those in control group. Our results were in agreement with a previous study reporting that metformin combined with CSII therapy needed less insulin titration time to achieve glycemic control than the control group, and 90% patients achieved euglycemic control by month 3^26^. Our data indicated that patients treated with a metformin add-on CSII therapy showed significant improvement in β-cell function as measured by HOMA-B and Insulinogenic Index. Although these are indirect measurements of beta cell function, they suggest a reason for the lower FPG and fasting insulin concentration found in the treated patients. In addition, patients treated with metformin combined with NPH therapy for 12-week had significantly decrease in MBG, MAGE, SDBG, and LAGE from baseline^27^.

CGM provides glucose concentrations every 5 min, which may be an option for determining 24-hr glycemic profiles. The increasing use of CGM leading to the better understanding of glycemic variability^31^, and promoting the emerging of treatment aimed at avoiding glucose fluctuations in diabetic patients^32^. A number of metrics obtained from CGM could be used to describe the GV in diabetic patients^14–16^. Using CGM data, clinical researchers and clinicians could efficiently evaluate the quality of the glycemic control which might be important for decision-making^20^. In this study, our CGM data revealed that patients in the metformin add-on CSII therapy group had further improvement in MAGE, SD, and CV compared with those in CSII alone therapy group. Studies have demonstrated that there was a high degree of correlation between SD and MAGE^13,18^. The percent CV displays well the interpretation of GV^13,18,19^. In addition, we observed that patients in the metformin add-on CSII group had lower glycemic variations during 0300–0600 o’clock compared with those in CSII alone group. In this study, the incidence of dawn phenomenon was 3.1% in all subjects. Our observation was in agreement of a previous study reporting that the dawn phenomenon affecting nearly 3% of T2D population^33^. However, our data could not address the underlying mechanisms of metformin decreasing the dawn phenomenon.

A trial conducted in subjects with type 1 diabetes indicated that a metformin add-on CSII therapy resulted in an improvement in blood glucose control and insulin requirements^21^. Of most importance, metformin combination with CSII is a safe therapy for the treatment of type 1 diabetes^21^. We previously observed that older (>60 years) men with newly diagnosed T2D had a high incidence of nocturnal low glucose before and during intensive insulin therapy, which indicates that special attention should be paid to prevention of hypoglycemia in elderly male patients when treated with intensive insulin therapy using an insulin pump^30^. In this study, we used insulin pump 712E (Medtronic Incorporated, Northridge, USA) delivered insulin in both groups, our data showed that patients treated with the metformin add-on CSII therapy had significantly decreased incidences of hypoglycemia (blood glucose <3.9 mmol/L). Although, we did not observe the long-run effect of metformin add-on CSII therapy on T2D glycemic control, the significant improvement in MAGE, SD, and %CV compared with control group indicating that metformin add-on CSII therapy may be the better option aimed at reducing glycemic fluctuations in T2D.

In the current study, we observed that drug naïve diabetic patients treated with metformin combination with CSII therapy experienced a significant reduction in the total daily insulin doses, bolus insulin doses, and basal insulin doses to maintain euglycemic control, the shorter times to achieve euglycemic control, the improvement in glycemic variation and associated with no increase of hypoglycemia, which indicates that metformin combined with CSII therapy can be considered a safe and valuable treatment option. However, the study patient population was limited to the Nanjing area in China, therefore, the situation might not be the same for other geographical regions or populations. The Chinese population had thrifty gene^34^, the high portion intake of carbohydrates and highly different life-style^35^. Moreover, Asian T2D populations have the lower BMI and smaller waist circumferences compared to the Western participants^36^. The better response to acarbose^37^, DPP-4 inhibitor^38^, GLP-1 receptor agonists^39^, and SGLT-2 inhibitor therapy^40^. These therapies have shown potential improvements in glucose fluctuation^37,38,40^ and lower insulin dose requirement by patients with T2D to maintain euglycemic control in Chinese populations^38^.

Our study has several limitations. Firstly, the study only observed patients population in Jiangsu Provence in China, so the situation might not be applicable to patients of other populations. Secondly, we did not observe the long-term effects of metformin add-on CSII therapy on glycemic control in patients with newly diagnosed T2D. Thirdly, we did not demonstrate the underline mechanisms of older men patients who had a higher risk of nocturnal hypoglycemia when treated using insulin intensive therapy. Lastly, a major limitation of study that performed in the inpatient setting with meals provided, therefore limited applicability to the real world.

In conclusion, our data showed that patients with newly diagnosed T2D may take advantage of the metformin add-on CSII therapy in requiring less insulin doses, and having available other methods of glycemic treatments in a hospital setting.

## Materials and Methods

The two studies were both randomized, controlled open-label trials. Between April 2012 and June 2016, we recruited a total of 188 patients with newly diagnosed T2D from Nanjing First Hospital in China. The inclusion criteria were (1) patients aged between >18 and <80; (2), HbA1c values >9.0% at diagnosis. Patients were excluded from analysis if they had serum creatinine levels ≥1.5 mg/dL (males), ≥1.4 mg/dL (females) or abnormal creatinine clearance, known hypersensitivity to metformin, acute or chronic metabolic acidosis, including diabetic ketoacidosis, with or without coma.

The study protocols and patient consent forms were approved by the Institutional Ethical Committee of Nanjing First Hospital. All patients gave written informed consent to participate. The methods were carried out in accordance with the Declaration of Helsinki guidelines, including any relevant details.

The trials included a screening period to collect baseline parameter values, a 2± weeks treatment period, and a CGM period. During the study period, all subjects were admitted as inpatients. Computer-generated random orders were prepared by the data-coordinating center and distributed to each patient to determine the patient’s treatment assignments. After the baseline parameters were assessed, subjects underwent oral glucose tolerance tests (OGTTs) using 75 g of glucose (dissolved in 200 ml water) before and after treatment. Serum samples were obtained before and 30, and 120 min after oral administration for glucose, insulin, C-peptide, and HbA_1c_ determination. Plasma insulin was determined using an insulin radioimmunoassay kit (Beijing Technology Company, Beijing, China). HbA_1c_ was measured by a DiaSTAT HbA_1c_ analyzer (Bio-Rad, Hercules, CA). C-peptide and glucose concentrations were measured centrally at the central laboratory in Nanjing First Hospital, Nanjing Medical University.

Enrolled subjects were then randomized either metformin add-on CSII or CSII only treatment. Metformin (Bristol-Myers Squibb, USA) was administered 500 mg thrice-daily. If the patients were unable to tolerate the minimum metformin dose (1000 mg/day), they were excluded from this study. The total daily insulin (Aspart, Novo Nordisk, Bagsværd, Denmark) doses were 0.4 IU/kg which was given in two injection modes: 1/3 of total daily dose was equally given as boluses within three meals, the remaining insulin was given as basal dose. Investigators titrated insulin doses on an individual-patient basis using the algorithm as before described^39^. When euglycemic control was achieved, treatments would remain unchanged for another 3 days. The treatment period required to achieve euglycemic control (the fasting capillary blood glucose was less than 6.1 mmol/L and capillary blood glucose at 2-hr after each of three meals was less than 8.0 mmol/L), were recorded for each subjects^41,42^. Changes in insulin doses following treatment were also analyzed.

All patients were subjected to 4-day retrospective CGM (Sof-sensor, CGMS-Gold, Medtronic Incorporated, Northridge, USA) in hospital by the specialist nurse after the completion of treatment. The CGM was performed by the study nurses, and the CGM data were saved by the investigator, as described previously^9,37,43^. During the CGM period, all subjects were instructed to maintain their usual physical activity and received meals consisting of a total daily caloric intake of 25 kcal/kg. The ratio of carbohydrate, proteins and fats was 55%, 17% and 28%, respectively.

The 24-hr mean glucose (MG), the SD, the CV, the MAGE, the incremental area under curve (AUC) of hyperglycemia (glucose >10.0 mmol/L) and hypoglycemia (glucose <3.9 mmol/L), the 2-hr postprandial after each meals, and the hourly BG were recorded and calculated. The β-cell function and insulin resistance were assessed by the homoeostasis model assessment B (HOMA-B) and HOMA-IR^42,44^. Matsuda Index^45^ and Insulinogenic Index^46^ were calculated as previously described.

The primary endpoints were differences in insulin doses and glycemic variations between groups at the completion of treatments. The times taken by the patients to achieve euglycemic control, the total daily insulin doses, the basal insulin doses, the bolus insulin doses, the hourly mean glucose concentrations, the 24-h BG and the AUC of hypoglycemia and hyperglycemia at the endpoint were also analyzed.

### Statistical analysis

All data were normal distribution and were presented as the means ± SD. Statistical significance was determined by one-way analysis of variance (ANOVA), or two-way ANOVA for repeated measurements for the group comparisons, followed by Bonferroni-Dunn *post hoc* test. P < 0.05 was considered to be statistically significant. All of the statistical analyses were performed using the Statistical Product and Services Solutions (SPSS) package (Version 11.5, SPSS, Science, Chicago, USA).
